# Salvage endoscopic nasopharyngectomy for recurrent nasopharyngeal carcinoma in a non-endemic area

**DOI:** 10.1007/s00405-024-08500-8

**Published:** 2024-03-14

**Authors:** Marco Valentini, Alessia Lambertoni, Giorgio Sileo, Alberto Daniele Arosio, Gianluca Dalfino, Fabio Pedretti, Apostolos Karligkiotis, Maurizio Bignami, Paolo Battaglia, Paolo Castelnuovo, Mario Turri-Zanoni

**Affiliations:** 1 Department of Otolaryngology Head and Neck Surgery, Department of Biotechnology and Life Sciences, ASST Lariana, Ospedale Sant’Anna, University of Insubria, 22042 Como, San Fermo Della Battaglia Italy; 2 Department of Otorhinolaryngology Head and Neck Surgery, Ospedale di Circolo e Fondazione Macchi, Department of Biotechnology and Life Sciences, ASST Sette Laghi, University of Insubria, 21100 Varese, Italy; 3 Head and Neck Surgery and Forensic Dissection Research Center (HNS&FDRc), Department of Biotechnology and Life Sciences, University of Insubria, 21100 Varese, Italy; 4 Department of Otolaryngology, University of Pavia, 27100 Pavia, Italy

**Keywords:** Endoscopic nasopharyngectomy, EBV, Nasopharyngeal carcinoma, Skull base, Head and neck oncology

## Abstract

**Purpose:**

To analyze oncological outcomes of endoscopic surgical treatment of locally recurrent EBV-related undifferentiated non-keratinizing nasopharyngeal carcinoma (uNK-NPC) in a non-endemic area.

**Methods:**

Retrospective review of patients affected by recurrent uNK-NPC treated with nasopharyngeal endoscopic resection (NER) in a tertiary-care referral center from 2003 to 2022, by evaluating survival rates, prognostic factors, and follow-up strategies.

**Results:**

The oncological outcomes of 41 patients were analyzed, over a mean follow-up period of 57 months. The 5-year overall, disease-specific, and disease-free survival of the cohort were 60.7% ± 8.9%, 69% ± 9%, and 39.7% ± 9.2%, respectively. The local (rT) and regional (rN) extension of recurrent disease, stage of disease, and status of resection margins appeared to significantly influence survivals. After a mean follow-up period of 21 months, a further recurrence after NER was observed in 36.6% of cases. Skull base osteonecrosis induced by previous irradiation and post-surgical bone remodeling represent the major challenges for early detection of further local relapses during postoperative follow-up.

**Conclusion:**

NER appeared as a safe and effective treatment for recurrent uNK-NPC. The adequate selection of patients eligible for NER is essential, to maximize the chances to cure and minimize the risk of local complications.

## Introduction

Nasopharyngeal carcinoma (NPC) is a malignant epithelial tumor with a peculiar geographical distribution (high incidence in Southeast Asia and North Africa) [1], a male gender prevalence (M:F -2.5:1) [2], and non-endemic in western countries where it accounts for 1–2% of all head and neck cancers [3]. According to histological features, NPCs are differentiated in keratinizing NPC, non-keratinizing NPC (differentiated and undifferentiated subtypes), and basaloid NPC [4]. The undifferentiated non-keratinizing form (uNK-NPC) has the higher incidence worldwide and shows a peculiar etiological culprit represented by the Epstein–Barr virus (EBV) latent infection. Markers specific for EBV infection (EBV encoded small RNAs, microRNA, and antibody against EBV antigens) are usually detected in patients affected by uNK-NPC [5–7]. Circulating levels of EBV-DNA represents an effective tool for monitoring disease progression, treatment efficacy, and disease recurrence [8–10]. Radiotherapy (RT) with or without concurrent chemotherapy (CT) is actually considered the gold standard treatment for primary disease, showing good results in term of oncological outcomes [11, 12]. Nevertheless, around 10% of patients present residual locoregional disease after primary treatment or experienced a local recurrence [13, 14]. Local recurrent NPC (rNPC) mostly is the expression of a radio-resistant cell population [15–17]. In addition, rNPC is surrounded by organs at risk that have already absorbed near tolerance radiation dose; hence, re-irradiation presents significant risk of toxicity [18–20]. For these reasons, surgical resection, whenever feasible, represents a valid treatment option for rNPC [21]. Salvage surgery has conventionally been performed through open approaches, but, since the first report in 2005 [22], endoscopic nasopharyngectomy (NER) has been gradually recognized as an appropriate treatment, especially as a consequence of its reduced invasiveness [23]. Endoscopic resection of the nasopharynx can be individually adapted and gradually extended according to the local extent of the tumor. NER type I is limited to the postero-superior nasopharyngeal wall, reaching the bony floor of the sphenoid sinus superiorly and the pharyngo-basilar/prevertebral fascia. NER type II is extended upwards to include the anterior wall and the floor of the sphenoid sinus. NER type III includes the removal of the lateral wall of the nasopharynx, the cartilaginous portion of the Eustachian tube, and the contents of the upper parapharyngeal space antero-medial to the internal carotid artery (ICA) [24]. Contraindications are massive intracranial intradural involvement, orbital apex invasion, cavernous sinus infiltration, and encasement of the ICA by the cancer. Recently, anecdotal cases of surgical resection of affected ICA have been reported, but the oncologic outcomes are still controversial [25, 26]. Different studies found that salvage surgery has similar survival outcomes to re-RT with decreased treatment-related morbidity and mortality [27–29]. Notably, NER still presents some critical issues: it is a challenging surgery, which may present intraoperative/postoperative complications, and postoperative surveillance could be insidious [29, 30].

The purpose of the present study is to present our experience in the management of locally recurrent EBV-related uNK-NPC in a non-endemic area, treated with NER, through the evaluation of the oncological outcomes, prognostic factors, and follow-up strategies.

## Materials and methods

### Study design

Patients with recurrent uNK-NPC after primary treatments (RT or RTCT) who were surgically treated at a single tertiary referral center from February 2003 to December 2022 were retrospectively enrolled in the study.

Inclusion criteria were as follows: (1) demographic, clinical, and follow-up data fully available; (2) salvage treatment by NER; (3) no evidence of systemic spread of disease before surgery; (4) follow-up of at least 12 months in living patients. The study was approved by the Institutional Review Board (Insubria Board of Ethics, approval number 0033025/2015). Informed consent was obtained from all subjects participating in the study.

### Workup, treatment, and follow-up

All cases were re-classified according to the 8th edition of the “TNM classification of malignant tumors” for nasopharyngeal cancer [31].

Details regarding the preoperative workup and NER surgical technique at the present institution have been extensively described in the previous papers [23, 24]. A concurrent (unilateral/bilateral) modified radical neck dissection (MRND) was performed when regional metastatic disease was suspected. Each case was fully discussed by the multidisciplinary tumor board and adjuvant treatments, such as re-irradiation or chemotherapy, were delivered in case of positive-resection margins or nodal metastasis with extracapsular extension (ECE).

Follow-up included endoscopic examination every 2 months and MRI with gadolinium every 4 months for the first year; endoscopic examination and MRI every 6 months until the 5th year, and clinical examination and MRI annually thereafter. Neck ultrasonography was performed every 6 months until the 5th year, then once a year. PET-CT was performed every year to rule out systemic spread of the disease. A quantitative test of peripheral blood levels EBV-DNA was performed annually. If local recurrence was suspected, multiple biopsies were taken endoscopically, under local anesthesia, when possible, otherwise under general anesthesia. Fine needle aspiration cytology was performed if regional recurrence was suspected.

### Statistical analysis

The main endpoints analyzed were overall survival (OS), disease-specific survival (DSS), disease-free survival (DFS), and local (RFST), regional (RFSN), and systemic (RFSM) recurrence free survival. The Kaplan–Meier method was used to estimate the probability of survivals with Greenwood standard errors and values were compared using the log-rank test. All statistical tests were two-sided, and *p *values were considered significant when ≤ 0.05.

The variables found to be significant in the univariate analysis were analyzed using Cox regression model; results were expressed as hazard ratio (HR), relative 95% confidence interval, and *p *values were considered significant when ≤ 0.05.

All analyses were performed using IBM SPSS Statistics^®^ software, version 25. (Chicago, IL, USA).

The sensitivity (SE) and specificity (SP) with related positive predictive value (PPV) and negative predictive value (NPV) were estimated for MRI, PET, and EBV-DNA plasmatic levels employed for the detection of local recurrence during postoperative follow-up.

## Results

### Demographic and clinicopathological characteristics

A total of 41 patients were enrolled in the study. Overview of demographic and clinicopathological data of the entire cohort is given in Table 1.

**Table 1:** Demographic, clinicopathological characteristics and treatment modalities

Characteristics	*N*	%	Characteristics	*N*	%
Sex			Margin status		
Male	28	68.3%	R0	34	82.9%
Female	13	31.7%	R1	7	17.1%
Age (years)			prT		
Median	50	/	1	19	46.3%
Range	31–81	/	2	12	29.3%
			3	8	19.5%
			4	2	4.9%
Stage of primary (AJCC 8th ed.)			prN		
I	4	9.7%	0	36	87.8%
II	8	19.5%	1	3	7.3%
III	22	53.7%	2	0	0%
IVa	7	17.1%	3	2	4.9%
IVb	0	0%			
Previous recurrences (before NER)			Postoperative complications		
None	26	63.4%	IMA bleeding (causing patient exitus)	1	2.4%
Local	4	9.8%	Conductive hearing loss	30	73.2%
Regional	7	17.0%	Trismus	24	58.5%
Local/regional	4	9.8%	Neck pain/cervical headache	21	51.2%
			Skull base osteonecrosis	16	39.0%
			Dysphagia/oro-nasal reflux	5	12.2%
Disease-free interval			Follow-up (months)		
Median	23,7	/	Median	57	/
< 24 months	27	65.9%	Range	12–139	/
> 24 months	14	34.1%			
NER			Recurrence after NER		
Type I	1	2.4%	None	26	63.4%
Type II	7	17.0%	Local	9	22.0%
Type III	33	80.5%	Regional	7	17.0%
			Systemic	3	7.3%
Surgical field resurfacing			Status		
None	14	34.1%	NED	22	53.7%
Nasoseptal flap	26	63.4%	AWD	3	7.3%
Temporoparietal fascia flap	1	2.5%	DOD	13	31.7%
			DOC	3	7.3%

*AJCC* American Joint Committee on Cancer, *AWD* alive with disease, *DOC* death of other causes, *DOD* death of disease, *NED* non-evidence of disease, *NER* nasopharyngeal endoscopic resection

Patients’ age ranged from 31 to 81 years (median, 50 years), with a male-to-female ratio of 2:1.

In 15 cases (36.6%), patients have already experienced a previous local/regional recurrence managed with a non-surgical protocol (re-RT or CT).

Time period from curative treatments (RT or CTRT) to NER was on average 23.7 months; time to recurrence was < 24 months in 27 cases (65.9%) and ≥ 24 months in the remaining 14 cases (34.1%).

Surgical procedures were classified as follows: NER type I in 1 case (2.4%), NER type II in 7 cases (17.0%), and NER type III in 33 cases (80.5%). Surgical field was resurfaced using a single or bilateral nasoseptal flap (26 cases) or a temporo-parietal fascia flap (1 case). A free-margin resection (R0) was obtained in 34 cases (82.9%), while in 7 cases (17.1%), microscopic positive margins (R1) were observed. Patients were submitted to MRND concurrent to NER in 6 cases (14.6%) and pathological nodal metastasis were confirmed in 5 cases (12.2%). Adjuvant therapy after surgical treatment was administered in 9 cases (21.9%): RT in 5 cases of R1; CT in 2 cases of R1 and 2 cases of ECE.

Major intraoperative complication occurred in one case of early postoperative massive bleeding from maxillary artery that resulted in death due to respiratory distress. Minor postoperative complications are reported in Table 1.

### Survivals analysis and prognostic factors

After a mean follow-up period of 57 months, 22 patients (53.7%) were alive without evidence of disease and 3 patients (7.3%) were alive with disease, while 13 patients (31.7%) died of disease and 3 patients (7.3%) of other causes.

The 3-year and 5-year OS of the entire cohort was 76% ± 7% and 60.7% ± 8.9%, respectively. The 3-year and 5-year DSS was 82.7% ± 6.5% and 69% ± 9%, respectively. The DFS was 52.7% ± 8.4% and 39.7% ± 9.2%, respectively.

The univariate analysis according to the different prognostic factors is reported in Table 2: local (rT) and regional (rN) extension of recurrent disease appeared to significantly correlate with prognosis in terms of OS (*p* = 0.003 and *p* = 0.004), DSS (*p* < 0.0005 for both parameters), and DFS (*p* < 0.0005 and *p* = 0.015), with better survival for early local recurrence and absence of neck nodes metastases.

**Table 2:** Univariate analysis of overall survival, disease-specific survival, disease-free survival local recurrence free survival, regional recurrence free survival, and systemic recurrence free survival according to prognostic factors

Variables		OS			DSS			DFS		
		3 years	5 years	*p* value	3 years	5 years	*p* value	3 years	5 years	*p* value
Sex	Male (28)	65.9% ± 9.3%	60.4% ± 10%	0.24	74.7% ± 9.0%	68.5% ± 10%	0.31	52.8% ± 10%	40,7% ± 10,8%	0.72
	Female (13)	100%	63.5% ± 16.9%		100%	71.4% ± 17.1%		51.9% ± 15.7%	34.6% ± 17.6%	
Stage at initial presentation	I (4)	100%	100%	0.67	100%	100%	0.23	100%	100%	0.25
	II (8)	83.3% ± 15.0%	83.3% ± 15.2%		83.3% ± 15.2	83.3% ± 15.3		52.2% ± 20.0%	N.A	
	III (22)	72.0% ± 9.7%	54.7% ± 11.5%		84.1% ± 8.4%	68.8% ± 12.0%		49.3% ± 11.4%	41.1% ± 12.1%	
	IVa (7)	71.4% ± 17.1%	53.6% ± 17.1%		71.4% ± 17.0%	53.6% ± 20.0%		42.9% ± 18.0%	21.4% ± 17.8%	
Stage at initial presentation merged	I + II (12)	88.9% ± 10.5%	88.9% ± 10.5%	0.31	88.9% ± 10.5%	88.9% ± 10.5%	0.3	68.8% ± 15.3%	51.6% ± 18.0%	0.48
	III + IV (29)	72.2% ± 8.4%	54.2% ± 10.1%		81.0% ± 7.7%	64.3% ± 10.5%		47.9% ± 9.7%	36.5% ± 10.2%	
Local recurrence pre surgical treatment	Yes (8)	62.5% ± 17.1%	62.5% ± 17.1%	0.23	62.5% ± 17.1%	62.5% ± 17.1%	0.13	62.5% ± 17.1%	31.3% ± 17.8%	0.87
	No (33)	80.0% ± 7.3%	59.6% ± 10.5%		88.9% ± 6.1%	70.0% ± 10.9%		49.4% ± 9.7%	43.9% ± 10.1%	
rT	1 (19)	88.5% ± 7.6%	88.5% ± 7.6%	0.003*	100%	100%	< 0.005*	70.6% ± 11.1%	62.7% ± 12.3%	< 0.005*
	2 (12)	71.3% ± 14.1%	57.0% ± 17.0%		77.8% ± 13.9%	77.8% ± 13.9%		42.9% ± 17.4%	N.A	
	3 (8)	75.0% ± 15.3%	37.5% ± 17.1%		75.0% ± 13.3%	37.5% ± 17.1%		37.5% ± 17.7%	25.0% ± 15.3%	
	4 (2)	N.A	N.A		N.A	N.A		N.A	N.A	
rN	0 (36)	78.9% ± 7.1%	62.2% ± 9.4%	0.004*	86.6% ± 6.3%	71.5% ± 9.5%	< 0.005*	55.1% ± 9.0%	40.2% ± 10.0%	0.015*
	1 (4)	66.7% ± 27.2%	66.7% ± 27.2%		66.7% ± 27.2%	66.7% ± 27.2%		50.0% ± 25.0%	50.0% ± 25.0%	
	3 (1)	N.A	N.A		N.A	N.A		N.A	N.A	
Stage at surgical treatment	I (16)	86.7% ± 8.8%	86.7% ± 8.8%	< 0.005*	100%	100%	< 0.005*	78.6% ± 11.0%	68.8% ± 13.3%	< 0.005*
	II (13)	82.1% ± 11.7%	68.4% ± 15.8%		88.9% ± 10.5	88.9% ± 10.5		48.5% ± 16.4%	24.2% ± 19.0%	
	III (8)	75.0% ± 15.3%	37.5% ± 17.1%		75.0% ± 15.3%	37.5% ± 17.1%		37.5% ± 17.1%	25.0% ± 15.3%	
	IVa (3)	N.A	N.A		N.A	N.A		N.A	N.A	
Stage at surgical treatment merged	I + II (29)	84.9% ± 7.0%	79.6% ± 8.3%	0.003*	95.7% ± 4.3%	95.7% ± 4.3%	< 0.005*	66.4% ± 9.8%	53.1% ± 11.5%	0.013*
	III + IV (11)	58.3% ± 14.2%	33.3% ± 13.6%		58.3% ± 14.2%	33.3% ± 13.6%		25.0% ± 12.5%	16.7% ± 10.8%	
Margin status	R0 (33)	80.2% ± 7.3%	67.1% ± 9.3%	0.08	88.9% ± 6.1%	78.1% ± 8.9%	0.005*	58.2% ± 9.3%	43.1% ± 10.2%	0.037*
	R1 (8)	57.1% ± 18.7%	N.A		57.1% ± 18.7%	N.A		31.3% ± 17.8%	N.A	
Time to recurrence	< 24 months (27)	76.6% ± 8.4%	70.7% ± 9.6%	0.23	86.8% ± 7.1%	80.2% ± 9.2%	0.065	62.8% ± 9.9%	51.4% ± 10.9%	0.085
	≥ 24 months (14)	75.0% ± 12.5%	42.9% ± 15.7%		75.0% ± 12.5%	50.0% ± 16.7%		31.2% ± 14.3%	15.6% ± 13.1%	

Variables		RFST			RFSN			RFSM		
		3 years	5 years	*p* value	3 years	5 years	*p* value	3 years	5 years	*p* value
Sex	Male (28)	60.9% ± 9.7%	54.1% ± 10.7%	0.27	90.7% ± 6.4%	82.4% ± 9.8%	0.18	83.2% ± 7.8%	83.2% ± 7.8%	0.47
	Female (13)	78.8% ± 13.4%	63.0% ± 17.7%		62.3% ± 15.0%	62.3% ± 15.0%		90.9% ± 8.7%	90.9% ± 8.7%	
Stage at initial presentation	I (4)	100%	100%	0.52	100%	100%	0.69	100%	100%	0.40
	II (8)	70.0% ± 18.25	N.A		83.3% ± 15.2%	83.3% ± 15.2%		N.A	N.A	
	III (22)	59.5% ± 11.1%	59.5% ± 11.1%		80.2% ± 10.3%	70.2% ± 13.0%		84.0% ± 8.5%	84.0% ± 8.5%	
	IVa (7)	71.4% ± 17.1%	35.7% ± 26.7%		64.3% ± 21.0%	64.3% ± 21.0%		68.6% ± 18.6%	68.6% ± 18.6%	
Stage at initial presentation merged	I + II (12)	80.2% ± 12.8%	60.2% ± 19.8%	0.70	88.0% ± 10.5%	88.0% ± 10.5%	0.45	100%	100%	0.15
	III + IV (29)	62.9% ± 9.4%	56.6% ± 10.3%		76.4% ± 9.4%	69.5% ± 10.8%		80.2% ± 8.0%	80.2% ± 8.0%	
Local recurrence pre surgical treatment	Yes (8)	62.5% ± 17.1%	31.3% ± 17.8%	0.17	100%	100%	0.14	72.9% ± 16.5%	72.9% ± 16.5%	0.27
	No (33)	67.5% ± 9.1%	67.5% ± 9.1%		74.4% ± 9.3%	67.6% ± 10.6%		88.7% ± 6.2%	88.7% ± 6.2%	
rT	1 (19)	82.4% ± 9.2%	74.1% ± 11.4%	< 0.005*	94.1% ± 5.7%	94.1% ± 5.7%	0.17	94.1% ± 5.7%	94.1% ± 5.7%	0.21
	2 (12)	66.7% ± 16.1%	66.7% ± 16.1%		74.1% ± 16.1%	37.0% ± 27.4%		90.0% ± 9.5%	90.0% ± 9.5%	
	3 (8)	50.0% ± 17.7%	33.3% ± 18.0%		53.6% ± 20.1%	53.6% ± 20.1%		62.5% ± 17.1%	62.5% ± 17.1%	
	4 (2)	N.A	N.A		N.A	N.A		N.A	N.A	
rN	0 (36)	71.4% ± 8.2%	60.6% ± 9.9%	0.10	78.1% ± 8.0%	72.5% ± 9.2%	0.70	86.8% ± 6.2%	86.8% ± 6.2%	0.65
	1 (4)	50.0% ± 25.0%	50.0% ± 25.0%		N.A	N.A		75.0% ± 21.7%	75.0% ± 21.7%	
	3 (1)	N.A	N.A		N.A	N.A		N.A	N.A	
Stage at surgical treatment	I (16)	85.7% ± 9.4%	75.0% ± 12.9%	< 0.005*	92.9% ± 6.9%	92.9% ± 6.9%	0.39	100%	100%	0.003*
	II (13)	69.3% ± 15.0%	69.3% ± 15.0%		77.1% ± 14.4%	51.4% ± 23.1%		90.9% ± 8.7%	90.9% ± 8.7%	
	III (8)	50.0% ± 17.7%	33.3% ± 18.0%		53.6% ± 20.1%	53.6% ± 20.1%		62.5% ± 17.1%	62.5% ± 17.1%	
	IVa (3)	N.A	N.A		N.A	N.A		N.A	N.A	
Stage at surgical treatment merged	I + II (29)	78.8% ± 8.4%	71.7% ± 10.3%	0.013*	87.0% ± 7.0%	79.1% ± 9.9%	0.45	96.0% ± 3.9%	96.0% ± 3.9%	0.01*
	III + IV (11)	41.7% ± 14.2%	31.3% ± 14.0%		61.0% 18.1%	61.0% 18.1%		59.5% ± 16.2%	59.5% ± 16.2%	
Margin status	R0 (33)	72.1% ± 8.4%	61.7% ± 9.9%	0.044*	83.7% ± 7.6%	77.7% ± 9.1%	0.17	85.4% ± 6.8%	85.4% ± 6.8%	0.83
	R1 (8)	46.9% ± 18.7%	N.A		64.3% ± 21.0%	N.A		87.5% ± 11.7%	N.A	
Time to recurrence	< 24 months (27)	75.4% ± 8.8%	69.6% ± 9.8%	0.089	90.8% ± 6.2%	83.2% ± 9.2%	0.095	82.4% ± 8.1%	82.4% ± 8.1%	0.52
	≥ 24 months (14)	47.5% ± 15.7%	31.7% ± 16.6%		57.0% ± 17.0%	57.0% ± 17.0%		92.3% ± 7.4%	92.3% ± 7.4%	

*DFS* disease-free survival, *DSS* disease-specific survival, *N.A.* not available, *OS* overall survival, *RFSM* systemic recurrence free survival, *RFSN* regional recurrence free survival, *RFST* local recurrence free survival

*Statistically significant values

**Fig. 1:**
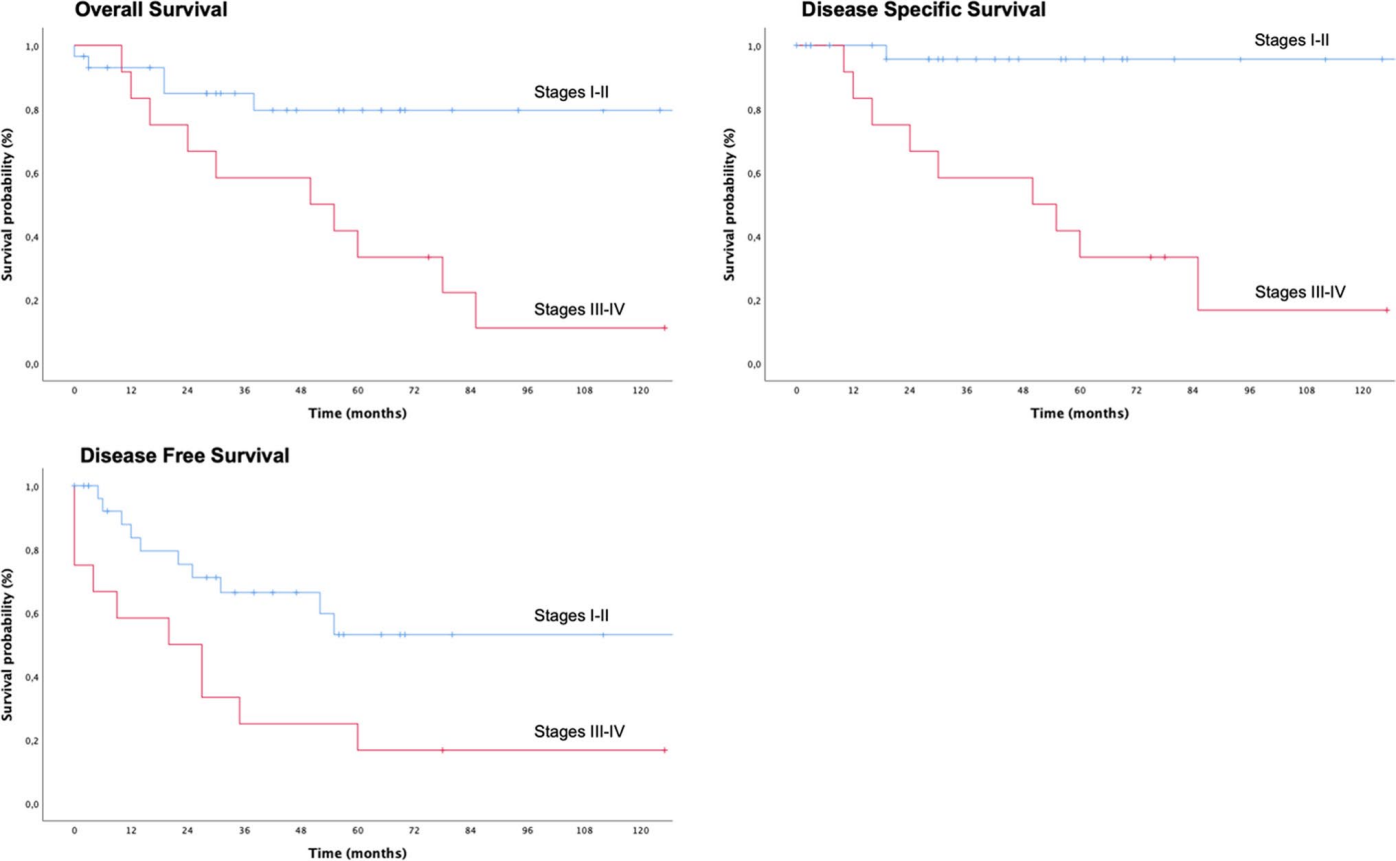
Overall survival (OS), disease-specific survival (DSS), and disease-free survival (DFS) stratifying patients according to tumor stage at the moment of surgical treatment

**Fig. 2:**
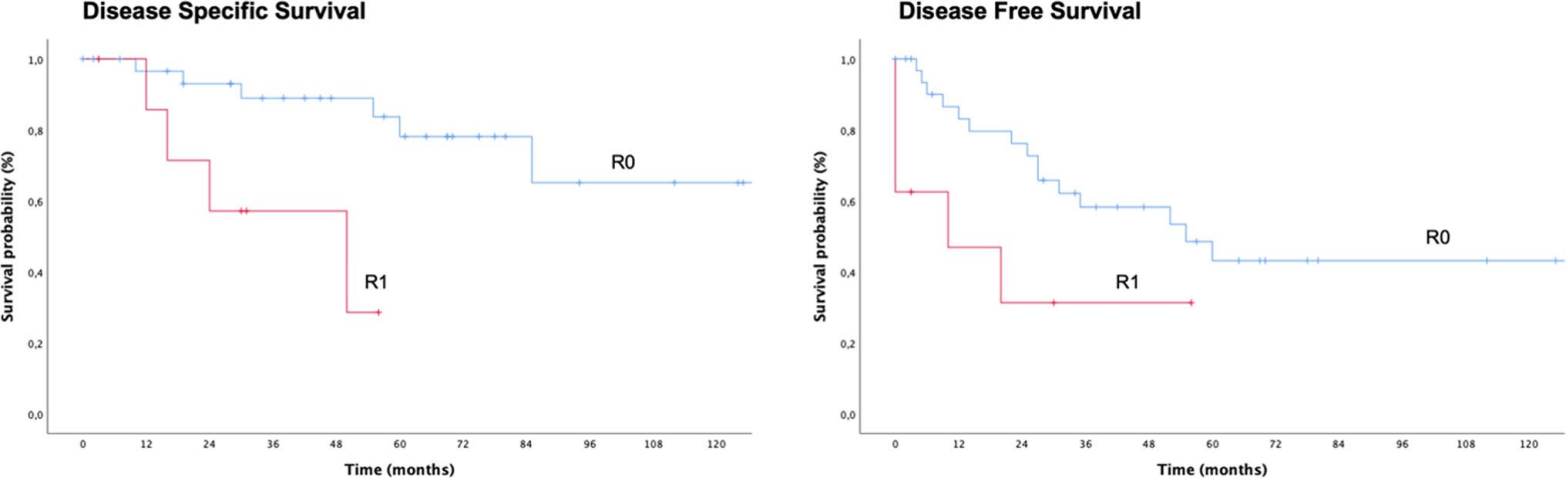
Disease-specific survival (DSS) and disease-free survival (DFS) stratifying patients according to status of resection margins

Similarly, the stage of disease significantly impacted on OS (*p* < 0.0005), DSS (*p* < 0.0005), and DFS (*p* < 0.0005) (Fig. 1): in detail, early stage tumors (stages I–II) showed a better survival when compared to advanced-stage tumors (stages III–IV). Patients with positive-resection margins showed worse survivals in terms of DSS and DFS compared to patients with a free-margin resection (*p* = 0.005 and *p* = 0.037, respectively) (Fig. 2). Gender, stage of primary tumor, previous local recurrence, and time to recurrence did not significantly impact on prognosis.

### Analysis of post-surgical recurrences

A total of 15 (36.6%) cases experienced further recurrences during the follow-up, as summarized in Table 1: six patients developed a further recurrence on T, three cases had a regional recurrence, two patients developed a systemic metastasis, two patients experienced simultaneous recurrence on T and N, one case had recurrence on N and M, and, finally, a simultaneous recurrence on T and M was observed.

These recurrences occurred after a mean period of 21 months after surgery (range 6–60 months).

Among them, 7 (46.7%) died of disease, 3 (20%) were alive with disease, 1 (6.6%) died of other causes, and 4 (26.7%) were alive without evidence of disease after treatments.

**Fig. 3:**
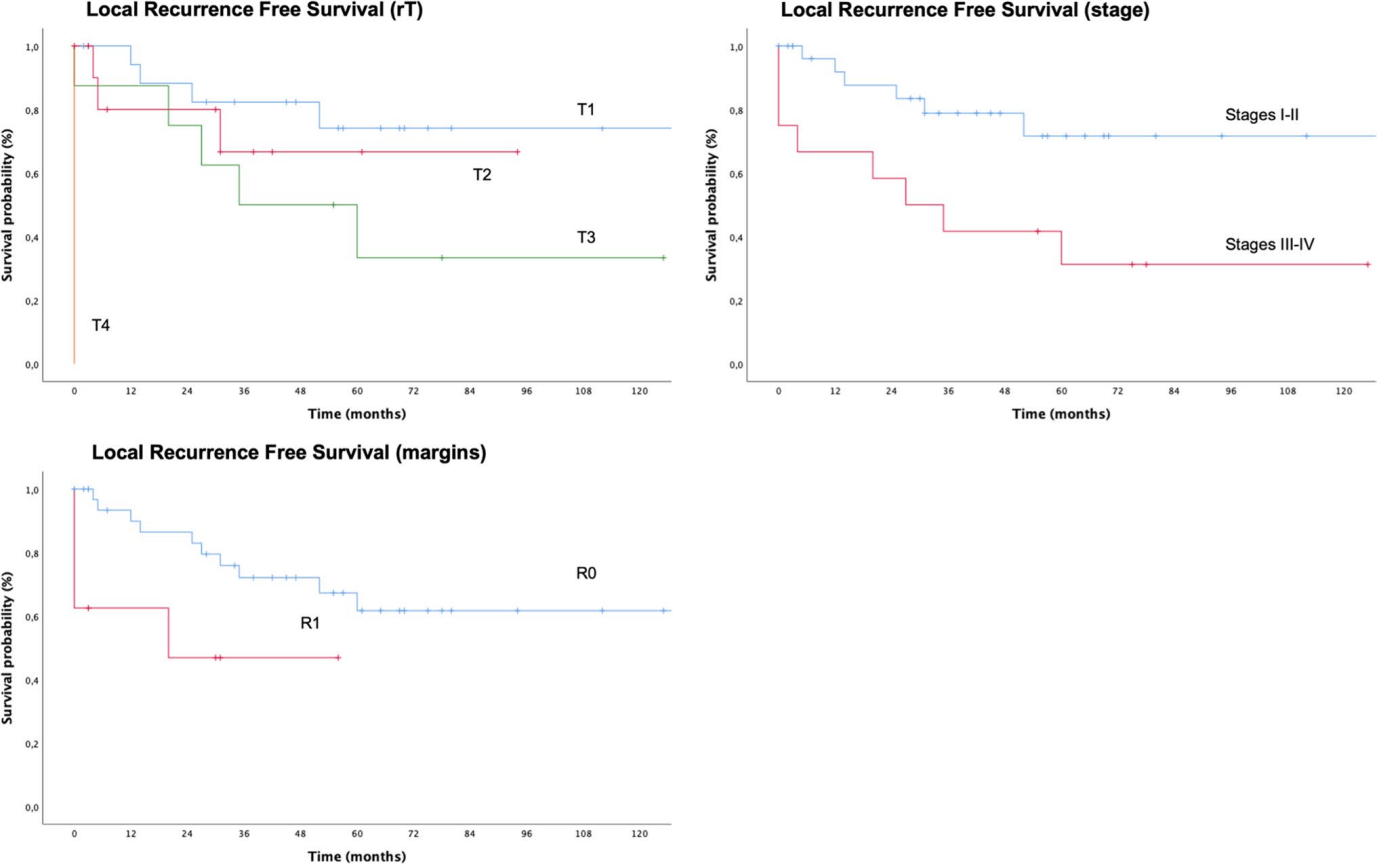
Local recurrence free survival (RFST) stratifying patients according to recurrence local extension (T), tumor stage at the moment of surgical treatment and status of resection margins (R)

The recurrence free survival on primary site (RFST) was 66.8% ± 7.9% and 57.4% ± 9.2% after 3 and 5 years, respectively. As shown in Fig. 3, the probability to experience an additional local recurrence depends significantly on local tumor extension and stage of disease at the time of NER (*p* < 0.005), and on the status of surgical margins (*p* = 0.037) (Table 2).

The RFSN was 79.8% ± 7.5% and 74.5% ± 8.7% after 3 and 5 years, respectively and did not correlate to any of the investigated variables (Table 2).

The probability to develop a systemic recurrence (RFSM) was considerably higher in case of advanced stages of disease (96% ± 3.9% for stages III–IV vs 59.5% ± 16.2% for stages I–II, *p* = 0.01). The RFSM was estimated to be 85.5% ± 6.1% after 3 and 5 years (Table 2).

### Multivariate analysis

On multivariate analysis (Table 3), the stage of disease (stage I–II vs stage III–IV) at the time of salvage surgery appeared to be an independent prognostic factor in terms of DSS (HR = 0.034, *p* = 0.034) and RFSM (HR = 9.85, *p* = 0.05). Moreover, a free-margin resection emerged as a protective factor in terms of DFS (HR = 2.99, *p* = 0.05). Age resulted to be an independent prognostic factor in terms of OS and DSS (*p* = 0.002 and *p* = 0.035, respectively).

**Table 3:** Multivariate analysis of overall survival, disease-specific survival, disease-free survival, local recurrence free survival, regional recurrence free survival, and systemic recurrence free survival

Variables	OS			DSS			DFS		
	HR	HR CI 95%	*p* value	HR	HR CI 95%	*p* value	HR	HR CI 95%	*p* value
Age	1.10	1.03–1.16	0.002*	1.09	1.00–1.18	0.035*	1.00	0.95–1.05	0.89
Stage at initial presentation merged	7.14	1.21–41.85	0.03*	8.70	0.76–99.71	0.082	1.45	0.41–5.08	0.55
Time to recurrence	1.12	0.32–3.84	0.85	1.32	0.25–6.85	0.74	1.59	0.61–4.11	0.33
Stage at surgical treatment merged	2.12	0.62–7.22	0.22	10.12	1.18–86.32	0.034*	2.40	0.90–6.38	0.079
Margin status	3.16	0.71–13.92	0.12	4.53	0.71–28.55	0.10	2.99	0.97–9.16	0.05*

*DFS* disease-free survival, *DSS* disease-specific survival, *HR* hazard ratio, *HR CI* hazard ratio confidence interval, *OS* overall survival

^*^Statistically significant values

### Follow-up

**Fig. 4:**
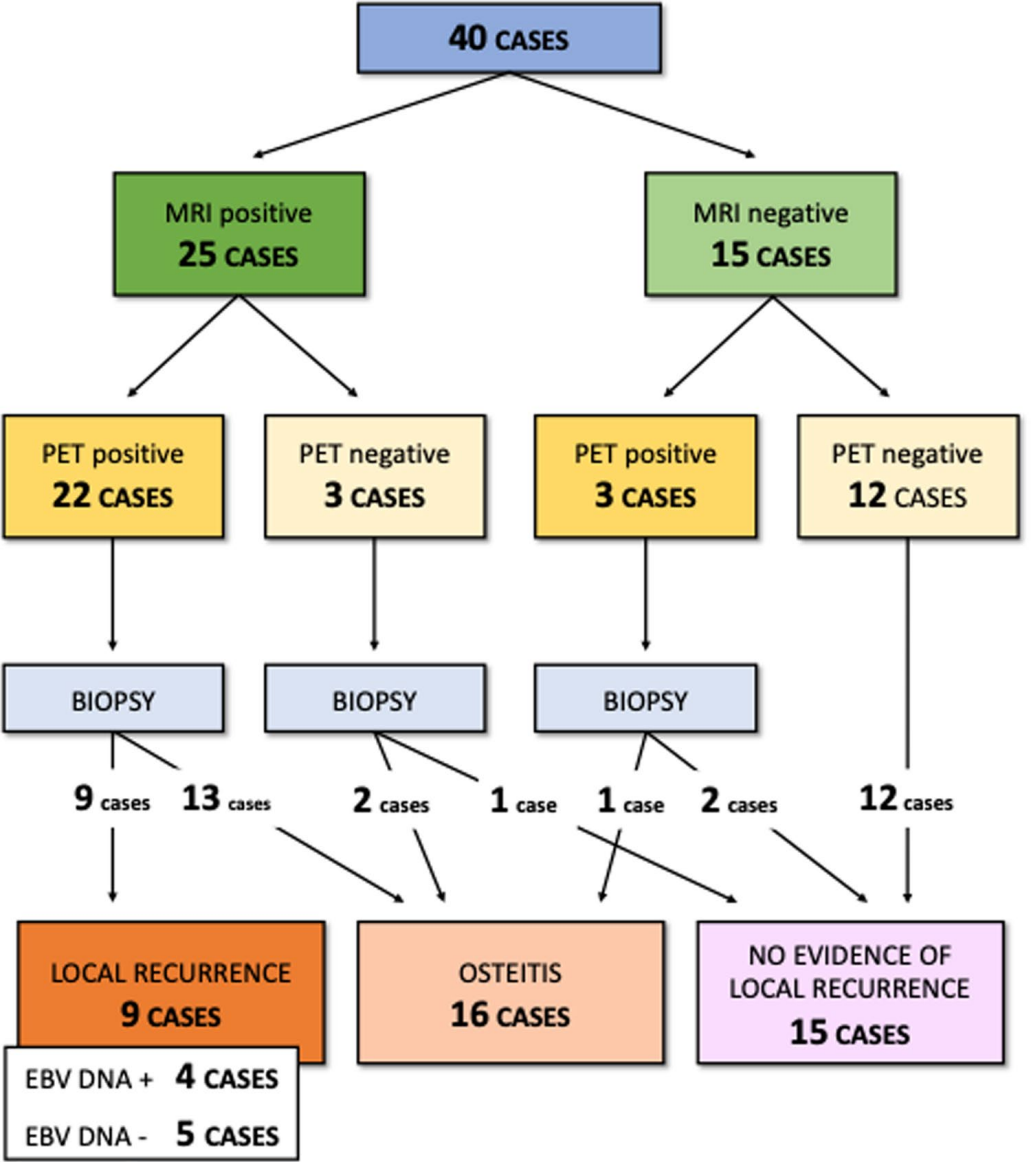
Flowchart indicating the results of examinations performed during follow-up

Follow-up analyses were conducted in 40 patients, since one patient died for complications in the early postoperative time. The follow-up period ranged from 12 to 139 months (mean, 57 months). The results of laboratory and radiological investigations performed during the follow-up are summarized in Fig. 4.

In 22 (55%) cases, MRI and ^18^FDG-PET were both compatible for suspect local recurrence; among these, the biopsy resulted positive for recurrence in 9 cases, while in the remaining 13 cases, the histological report documented chronic inflammation and/or necrotic bone tissue compatible with osteonecrosis.

In 12 (30%) cases, MRI and ^18^FDG-PET were both negative for suspect local recurrence: in these cases, no biopsies were needed.

In 6 (15%) cases, only one investigation (MRI or ^18^FDG-PET) was positive: in all such cases, a biopsy was taken, but no local recurrence was histologically documented.

Increased levels of plasmatic EBV-DNA were observed in five cases (12.5%): in four cases, patients were affected by local recurrence, and in one case, a systemic recurrence was observed.

Globally, the radiological exams (MRI and ^18^FDG-PET) showed a sensitivity of 100%, but limited values of specificity (MRI = 48%, ^18^FDG-PET = 50%); conversely, the plasmatic EBV-DNA had a low sensitivity (44%) and high specificity (96%).

## Discussion

The results emerging from the present study support the role of NER as safe and effective treatment option for recurrent uNK-NPC. Appropriate selection of patients and adequate surgical resection based on tumor extent represent the paramount issues in this regard. In our experience, an NER type III (laterally extended to include the parapharyngeal space and cartilaginous portion of the Eustachian tube) was mostly performed (80.5%), although recurrences were early staged (rT1 and rT2) in the majority of cases (75.6%). This could be explained considering that the main goal of salvage surgery should be a free-margin resection, and therefore expanded approaches are generally preferred. In our series, locally advanced recurrences (rT4) have been surgically treated in two cases, even if such tumor extension is considered as a contraindication for salvage surgery. In such cases, probably, the local recurrence have been under staged on preoperative examination, due to the limited imaging capabilities in accurately determining tumor local extension when embedded in post-irradiation scar and fibrosis. In these cases, the result was a positive margin surgical resection, emphasizing the importance of accurate preoperative analysis.

In this regard, the statistical analyses confirmed that rT stage and surgical margin status appeared to significantly influence the survival in our series, in accordance with current literature [13, 32, 33]. In detail, Chan et al. showed that the chance of local recurrence after NER was 10.7%, 38.5%, and 67.7% in patients with clear (at least 5 mm), close (< 5 mm), and involved resection margins, respectively [33].

In literature, the stage of primary disease and the time to recurrence have been described as prognostic factors, since patients with advanced-stage tumors who experienced an early recurrence showed decreased survival rates [30]. Conversely, from our results, the stage of primary tumor and the time to recurrence were not associated with statistically significant values.

Considering concurrent regional recurrence, our analysis reveals reduced survival rates (OS, DSS, and DFS) proportional to the degree of nodal involvement. To note, extracapsular extension (rN3) appeared to significantly impact on survival, as confirmed by numerous studies available in the literature [34–39]. However, these statistical data should be taken with caution in view of the small number of cases (5 cases). When extranodal extension or multiple pathologically positive lymph nodes are present, adjuvant chemotherapy may potentially reduce the risk of distant relapse eradicating micro-metastases, while for cases with isolated suspicious node (rN1) located at level II or III nodal basin, a selective neck dissection could be sufficient, to reduce treatment-related morbidity [35, 40, 41].

In the current literature, data regarding incidence and treatment of further recurrences following NER are lacking. The survival is generally poor for this cluster of patients, as inferable from our experience. All the documented further recurrences (36.6% of the whole series) were detected within 5 years from NER. Survival rates for these patients are poor, and most of them have died of the disease within 12 months or are alive but with disease. The risk to develop additional local recurrence is statistically determined by the same overmentioned prognostic factors (stage of disease and surgical margins in univariate analysis, RFST), underlining that obtaining a complete excision is crucial for survival.

NER is a high-risk procedure burdened by potentially life-threatening intraoperative complications, including ICA blowout and death [28]. Furthermore, patients may experience long-term postoperative minor sequelae significantly affecting their quality of life (e.g., nasal crusting, trismus, dysphagia, and conductive hearing loss), related also to previous irradiation. In selected cases of severe postactinic masticatory dysfunction requiring extensive nasopharyngectomy at high vascular risk, protective tracheostomy should be considered. In addition, the wide area of exposed bone resulting from NER might undergo an incomplete and delayed healing, with consequent occurrence of postoperative wound infection, clival and middle skull base osteomyelitis or osteonecrosis, which can even cause delayed ICA blowout [42–46]. According to our experience, prolonged postoperative bone inflammation was a common finding, sometimes requiring surgical debridement in case of necrosis progression. To prevent such sequelae, the surgical field should be resurfaced by local or regional flaps, as described by many authors in the literature [29, 30, 47–50].

**Fig. 5:**
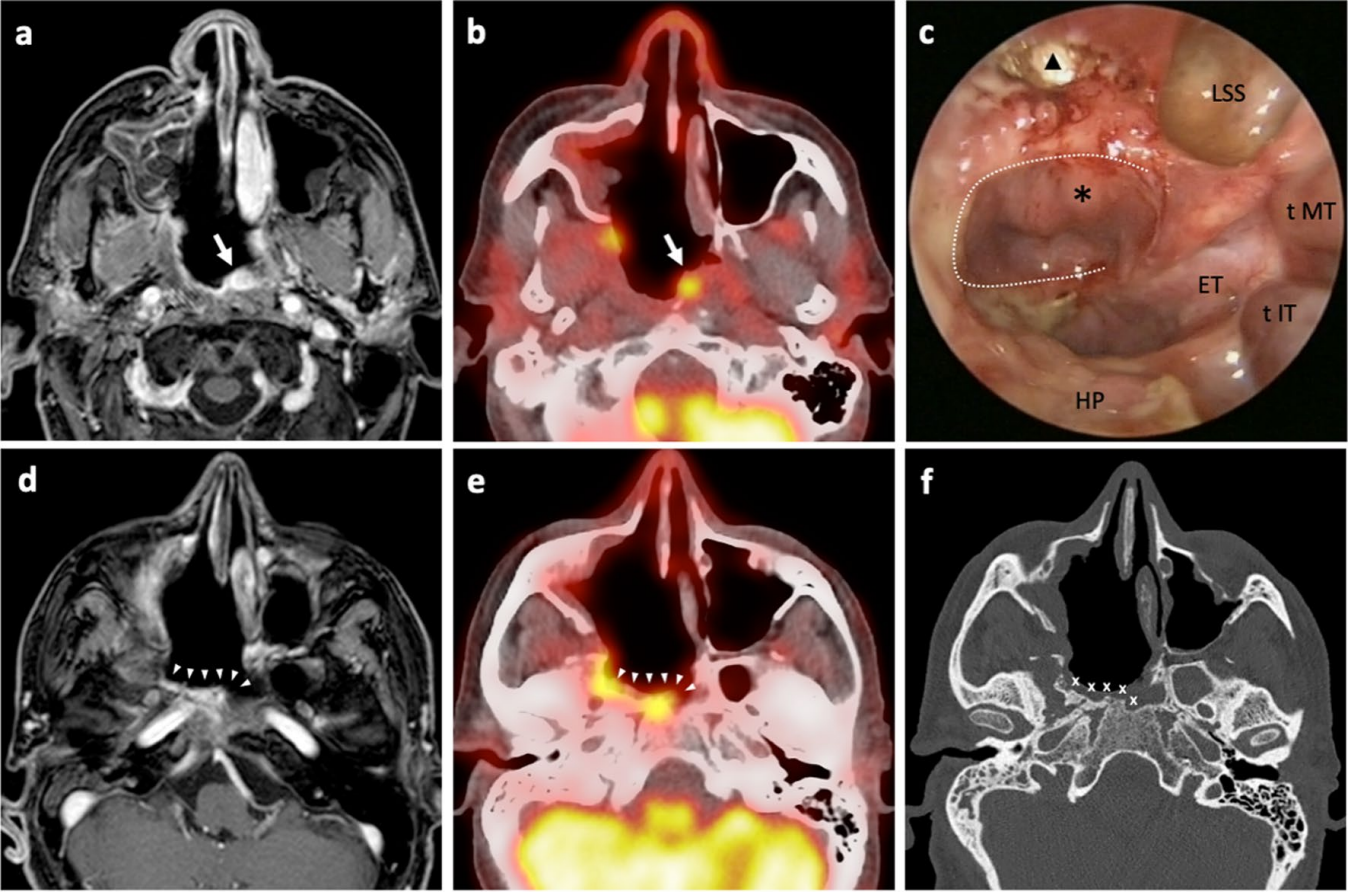
Radiological and clinical follow-up performed nine months after NER type 3 (right side) and ipsilateral MRND for recurrent uNK-NPC (rpT1N3cM0) in a 48 year old patient. An area of focal contrast-enhancement (**a**) and ^18^FDG uptake (**b**) was evident at the level of the left portion of the nasopharynx (white arrow), corresponding at the endoscopic evaluation (**c**) to a small swelling (black asterisk) below the left pedicled nasoseptal flap (white dotted line). Imaging investigations showed another area of diffuse contrast-enhancement (**d**) and ^18^FDG uptake (**e**) at the level of the right pterygoid area (white arrowheads) which, at the endoscopic evaluation (**c**), appears as an area of exposed necrotic bone and granulations (black triangle). Biopsies were taken from both areas of suspect signal, resulting in local recurrence of uNK-NPC at the level of the left nasopharynx and chronic inflammation at the level of right pterygoid, due to underlying osteonecrosis (white crosses) as demonstrated by CT scan (**f**). *ET* Eustachian tube, *HP* hard palate, *LSS* left sphenoid sinus, *tIT* tail of inferior turbinate, *tMT* tail of middle turbinate

Follow-up of patients with NPC should include early detection of tumor recurrence and assessment of delayed adverse events. According to the Chinese Society of Clinical Oncology [12], follow-up methods include endoscopic evaluation, nasopharyngeal and neck MRI, and serum EBV-DNA load detection. A radiological differential diagnosis between local recurrence and treatment-related inflammation may be challenging [51, 52]. In our experience, a false-positive signal with controversial radiological findings was detected on MRI in more than half of the cases. To overcome this problem, some authors suggest the use of ^18^FDG PET, even if such exam is burden by some limitations, as well (Fig. 5) [51]. According to this series, no significative differences in terms of specificity among these techniques have been observed (MRI = 48% versus ^18^FDG PET = 50%). In addition, some authors proposed the use of EBV-DNA levels to diagnose recurrency based on the reported high sensitivity (0.85) and specificity (0.89) [53]. However, data emerging from this series, and in line with the other reports, showed that less than half of recurrences presents elevated serum EBV-DNA load [54]. In our opinion, therefore, all three investigations should be included in the follow-up strategy, since each exam does not supersede the role of the others. Considering the difficulty of obtaining a correct differential diagnosis using radiologic imaging and EBV-DNA load, we believe that a histologic examination is still the most reliable procedure to confirm a possible local recurrence, whenever feasible.

The present study has some limitations that cannot be neglected. First, it is based on a retrospective analysis of cases over a 20-year period, which might have introduced biases related to changes in staging systems and treatment modalities. Second, it is based on a small population with significative impact on statistical analysis relevance. However, it is important to underline that although small if compared to studies carried out in endemic areas, it represents one of the largest experiences on salvage surgery for rNPC in a non-endemic area. Third, the population is limited to cases amenable to surgical salvage treatment, and therefore, it is mainly composed by early stage local recurrences, configuring a selection bias.

In conclusion, the surgical management of rNPC is often challenging considering the anatomical complexity of the region, previous irradiation, and the significant rate of possible complications. Patients’ survival is mainly affected by the ability to reach a radical tumor resection, which represents the essential goal of salvage surgery. On this regard, we believe that proper selection of patients eligible for salvage NER represents the crucial aspect in the management of rNPC as incomplete tumor resection, with the potential necessity of further oncological treatments, carries the risk of severe complications, not outweighed by an increase in survival outcomes. Indeed, we strongly recommend performing salvage endoscopic surgery in a single modality treatment setting to maximize the chance of cure for these exceptionally fragile patients.

## Conclusions

Local failures remain one of the greatest challenges in the management of NPC. NER has been proven to be a reliable and effective treatment, although recurrent NPC might present severe prognosis. The adequate selection of patients eligible for NER is crucial to maximize the survival outcomes and minimize complications rates.

Follow-up is aimed to either early detect further relapses or assess late-onset treatment-related sequelae. Nonetheless, differential diagnosis appeared to be challenging, and thus, in the majority of cases, biopsy collection and histological examination should be suggested.

## Data Availability

Not applicable.
